# Significant ophthalmoarthropathy associated with ectodermal dysplasia in a child with Marshall-Stickler overlap: a case report

**DOI:** 10.1186/1757-1626-1-270

**Published:** 2008-10-24

**Authors:** Ali Al Kaissi, Rudolf Ganger, Klaus Klaushofer, Franz Grill

**Affiliations:** 1 Ludwig Boltzmann Institute of Osteology, at the Hanusch Hospital of WGKK and, AUVA Trauma Centre Meidling, 4th Medical Department, Hanusch Hospital, Vienna, Austria; 2 Orthopaedic Hospital of Speising, Paediatric Department, Vienna, Austria

## Abstract

**Background:**

The Marshall-Stickler phenotype is an autosomal dominant trait comprising ocular abnormalities, sensorineural hearing loss, craniofacial anomalies, and anhidrotic ectodermal dysplasia.

**Case presentation:**

A 5-year-old boy from non-consanguineous family in Austria was born with features of Pierre-Robin association (cleft palate, micrognathia, and glossoptosis). Radiological examination at birth revealed coronal clefts of the vertebrae, platyspondyly, and flaring of the metaphyses of the long bones (features suggestive of the Weissenbacher-Zweymuller syndrome). Significant features of ectodermal dysplasia such as sparse hair, defective dentition, dysplastic nails, and deficient sweating associated with bouts of unexplained hyperthermia were present. These features not shared by Stickler syndrome, Wagner syndrome, or Weissenbacher – Zweymuller syndrome, all of which are conditions often confused with Marshall syndrome.

**Conclusion:**

There is continuing debate over the clinical overlap and differential diagnosis of Marshall and Stickler syndromes. We compared similar disorders, such as Weissenbacher-Zweymuller, and Wagner syndromes, and conclude that our present patient manifests Marshall-Stickler overlap. Focussing on subtle facial and ectodermal features may detract from recognising the serious outcome of congenital vitreous/myopia anomaly. Retinal detachment with subsequent blindness is a major risk in our current patient.

## Background

The nosologic relationship of the Stickler and Marshall syndromes and the nosologic relationship of the Marshall and Wagner syndromes have been much mooted [1-6]

Ayme and Preus [7] Suggested that the facies differ. Patients with the Marshall syndrome have a flat or retracted midface whereas those with the Stickler syndrome have a flat malar, which is often erroneously described as a flat midface. Baraitser [8] suggests that Stickler syndrome is identical to Marshall syndrome, although this is still disputed by Ayme and Preus. Marshall syndrome patients have a significant short stature, thick calvaria, abnormal frontal sinuses, and intracranial calcifications. The eyeballs appear large, possibly because of a shallow orbit. Marshall [3] emphasized the ectodermal abnormalities, including defects in sweating and dental structures in the family he reported. Ectodermal dysplasia and ocular hypertelorism, features not shared by Stickler syndrome, Wagner syndrome, or Weissenbacher – Zweymuller syndrome, all of which are conditions often confused with Marshall syndrome [1-6].

## Case presentation

A 5-year-old boy was referred to the department of paediatric orthopaedics because of large/prominent and painful joints. The child was a product of full term uneventful gestation. At birth his weight, length and OFC were around the 10 Th percentile. Features of Pierre-Robin association have been identified. A median cleft palate, micrognathia, and glossoptosis were the main prominent features. The mother was a 29-year-old gravida 1 abortus 0 married to a 33-year-old unrelated man. Both were healthy and their family history was unremarkable. We performed complete physical examination for the parents, which include full oro-facial, musculo-skeletal, ophthalmologic, and audiologic assessments. No relevant features have been detected.

Nasopharyngeal cannulation has been arranged to overcome his respiratory obstruction. This cannula, which itself acts as an airway, primarily acts as a sort of "splint" which makes further airways on either side of the tube between the tongue and the throat wall, thus assisting the infant in breathing and preventing the tongue from falling back down into the throat, which would cause the infant to asphyxiate. This treatment was carried out for 6 months, at a time the jaw showed sufficient growth. His cleft palate has been repaired at the age of 8 months. His glossoptosis and micrognathia did not require surgical intervention, as they had improved, but his mandibular arch remains significantly smaller than average. Sensorineural heraing loss has been detected at the age of 3 years. Bilateral hearing aids and a speech and language therapist and audiologist have been assigned later on to assist the child's speech retardation. His subsequnet course of motor development has been of significant retardation. Early joint stiffness associated with painful bouts of early osteoarthritis were additional burden and involved the weight bearing joints(hip, knee and ankle joints). Early lumbar spondylosis associated with early osteoarthritis of the neck and the back were present. Walking has been achieved lately albeit with difficulty because of the enlarged/stiff joints, superimposed by his defective vision. He had a history of frequent bouts of unexplained hyperthermia. Examination showed growth deficiency of -4 SD. Craniofacially, there was brachycephaly, sparse skull hair, a retracted mid face, faint eyeborws, sparse eye lashes, ocular hypertelorism (the eyeballs appear large) and hypodontia was evident. The skin is dry and the nipples are hypoplastic. The nails showed significant dysplasia. He wore bilateral hearing aids and had a high-grade myopia of – 8 diopters. The routine blood and urine tests, serum electrolytes and rheumatoid tests (C-reactive protein, ESR, rheumatoid factor) were negative. Serum and urinary oligosaccharides, mucopolysaccharides, serum lactate, pyruvate, creatine phosphokinase, and chromosomal study were normal.

**Figure 1:**
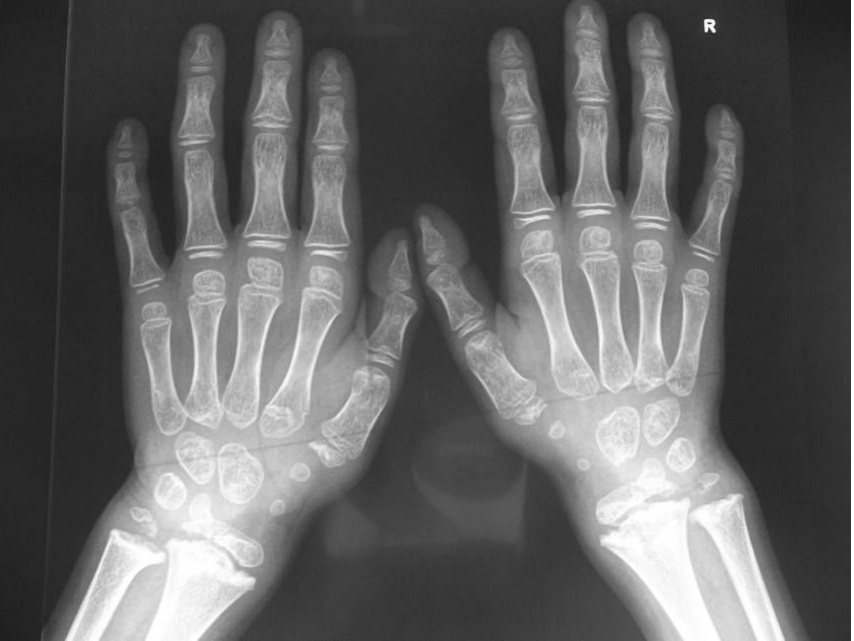
Anteroposterior hand radiograph showed, short-stubby hand with large carpal bones associated with pseudoepiphyses of the second proximal metacarpophalangeal joint.

**Figure 2:**
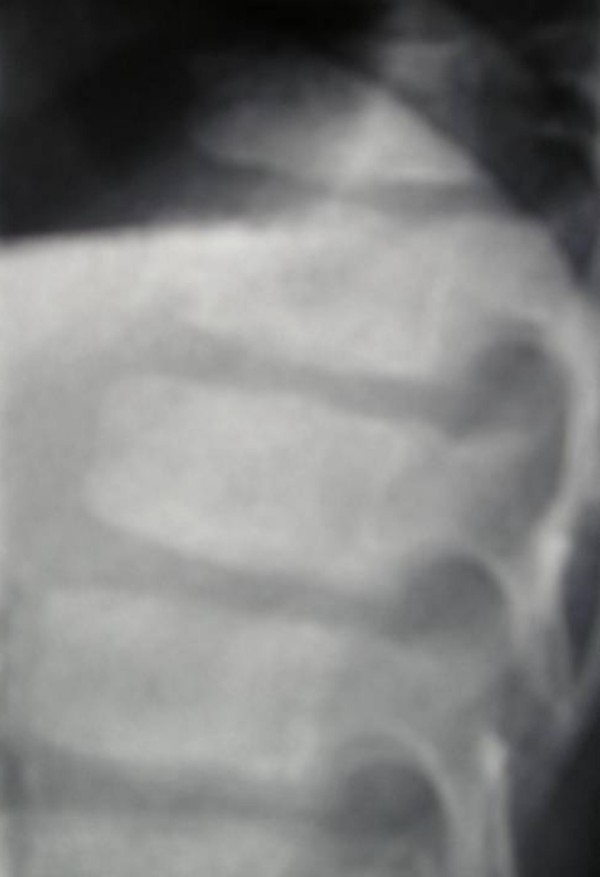
Lateral spine radiograph showed platyspondyly associated with anterior clefting of the vertebrae.

**Figure 3:**
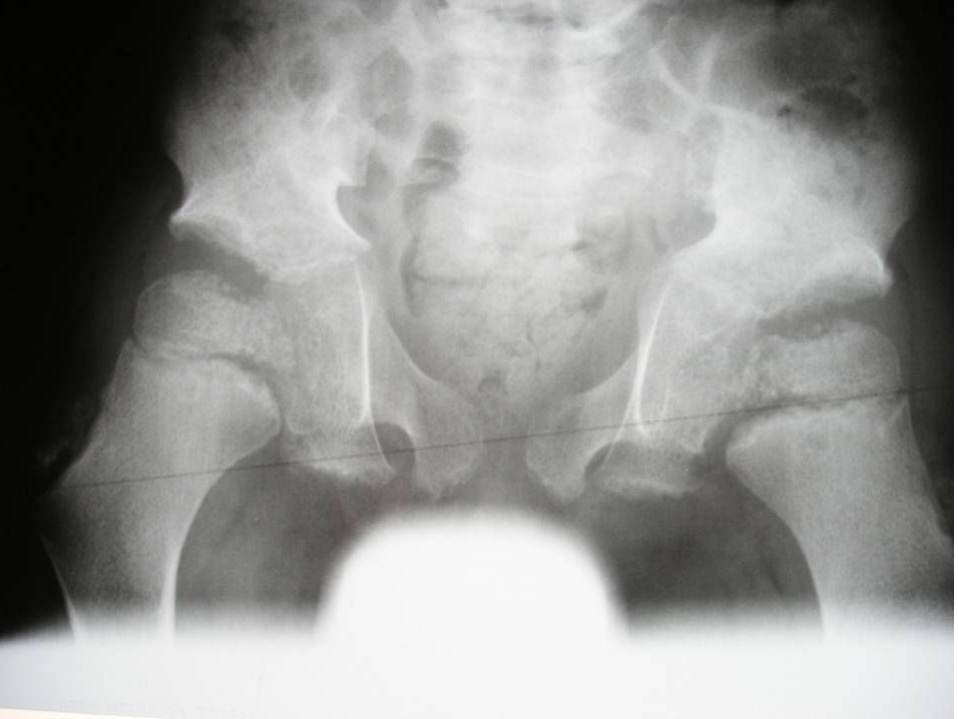
**Anteroposterior pelvis radiograph showed, coxa valga, a hypoplastic ileae, acetabulo-femoral and metaphyseal dysplasia, with the propensity for the development of slipped epiphyses associated with multiple epiphyseal fragmentations/stippling.** The latter might lead for the development of Perthes disease. Early signs of early osteoarthritis were evident.

On the bases of the skeletal survey, lateral skull radiograph showed absent frontal sinus, mid-facial hypoplasia and relative thickening of the calvaria. Anteroposterior hand radiograph showed, short-stubby hand with large carpal bones associated with pseudoepiphyses of the second proximal metacarpophalangeal joint (fig 1). Lateral spine radiograph showed platyspondyly associated with anterior clefting of the vertebrae (fig 2). Anteroposterior pelvis radiograph showed, coxa valga, a hypoplastic ileae, acetabulo-femoral and metaphyseal dysplasia, with the propensity for the development of slipped epiphyses associated with multiple epiphyseal fragmentations/stippling. The latter might lead for the development of Perthes disease. Early signs of early osteoarthritis were evident (fig 3). Knee radiographs showed significant epi-metaphyseal dysplasia associated with the development of cupped-metaphyses of the distal tibio-fibular. A picture of ball and socket ankle joint associated with features of early osteoarthritis.

## Discussion

Approximately half of all individuals with Robin sequence have an underlying syndrome, of which Stickler syndrome is the most common. In one study, 34 of 100 individuals with Robin sequence had Stickler syndrome [9]. Van den Elzen et al., [10] found that 15% of a series of children with Pierre Robin association had Stickler syndrome. Holder-Espinasse et al., [11] also found that about 15% of cases presenting at birth with Pierre-Robin syndrome had Stickler syndrome. Radiological examination at this time may reveal coronal clefts of the vertebrae, mild platyspondyly and flaring of the metaphyses of the long bones (features of the Weissenbacher-Zweymuller syndrome). As the child grows older these features become normal, although a mild epiphyseal dysplasia may develop. Weissenbacher-Zweymuller syndrome has been described as "neonatal Stickler syndrome," but is actually a distinct entity [12]. It is characterized by midface hypoplasia with a flat nasal bridge, small upturned nasal tip, micrognathia, sensorineural hearing loss, and rhizomelic limb shortening. Radiographic findings include dumbbell-shaped femora and humeri and vertebral coronal clefts. The skeletal findings become less apparent in later years, and catch-up growth after age two to three years is common [5].

Stickler syndrome (hereditary arthro-ophthalmopathy) first recognised by Stickler et al., [1,2] in a family with ocular, orofacial, auditory, and musculoskeletal abnormalities. Variants of Stickler syndrome have been designated STL1 (vitreous type with mutations in COL2A1 gene), STL2 (early onset hearing loss and mutations in the COL 11A1 gene) and STL3 (non-ocular type caused by mutations in the COL11A2 gene). Numerous genetic mutations of Stickler syndrome cases are STL1 variety and are caused by mutations in the COL2A1 gene. In practice, however, there are difficulties in obtaining molecular analysis because of the size, complexity and the number of gene involved and the high cost of these investigations. Francomano et al., [13] reported close linkage to COL2A1 in several families. COL2A1 mutations have been reported in some families, including the original Stickler kindred [14]

Individuals with Marshall syndrome manifest ocular hypertelorism, hypoplasia of the maxilla and nasal bones, flat nasal bridge, and small upturned nasal tip. Marshall [3] reported 4 generations of a family in which 7 members had, nasal defect and facies characteristic of anhidrotic ectodermal dysplasia, congenital and juvenile cataracts, myopia and fluid vitreous, spontaneous, sudden maturation and absorption of congenital cataract, luxation of cataract and congenital hearing loss. The transmission was dominant. In contrast to Stickler syndrome, the flat facial profile of Marshall syndrome is usually evident into adulthood. Radiographs demonstrate hypoplasia of the nasal sinuses and a thickened calvarium. Ocular manifestations include high myopia, fluid vitreous humor, and early-onset cataracts (subcapsular, cortical, nuclear, zonular, or anterior axial embryonic sites). Sensorineural hearing loss is common and can be progressive. Cleft palate, both as part of the Pierre Robin sequence and as an isolated anomaly, is seen. Other manifestations include short stature and early-onset arthritis. Skin manifestations include mild hypotrichosis and hypohidrosis [5,6,15]. Splice site mutations in the *COL11A1 *gene have been identified by Griffith et al [15]

## Conclusion

Both Stickler syndrome and Marshall syndrome are dominantly inherited chondrodysplasias characterized by midfacial hypoplasia, high myopia, and sensorineural hearing deficit. Since the characteristics of these syndromes overlap, it has been argued whether they are distinct entities or different manifestations of a single syndrome. The majority of individuals with Stickler/Marshal syndrome have inherited the mutant allele from a parent. On the light of no similar conditions in the family we might presume that our patient had developed the disorder as a result of a de novo gene mutation. Genetic counselling in this family looks difficult to accomplish since molecular genetic testing is usually not offered in the absence of a known disease-causing mutation in a parent. Finally we might propose that phenotypic overlap exists to suggest that Marshal and Stickler syndrome are probably allelic expressions of the same locus.

## Abbreviations

SD: Standard deviation; ESR: Erythrocyte sedimentation rate; STL 1: Stickler syndrome type 1 and so forth; COL2A1: Collagen 2 alpha 1 and so forth.

## Consent

Written informed consent was obtained from the parents for the purpose of publication of the manuscript and figures of their child. A copy of the written consent is available for review by the editor-in-Chief of this journal.

## Competing interests

The authors declare that they have no competing interests.

## Authors' contributions

All of the authors were involved in the clinico-radiographic assessment and finalising the paper. All authors have red and approved the final version of the paper.
